# Long-Term Variations in Retinal Parameters after Uncomplicated Cataract Surgery

**DOI:** 10.3390/jcm11123426

**Published:** 2022-06-15

**Authors:** Maciej Gawęcki, Natalia Prądzyńska, Izabella Karska-Basta

**Affiliations:** 1 Department of Ophthalmology, Specialist Hospital in Chojnice, 89-600 Chojnice, Poland; 2 Dobry Wzrok Ophthalmological Clinic, 80-280 Gdańsk, Poland; natdor1995@wp.pl; 3 Department of Ophthalmology, Faculty of Medicine, Clinic of Ophthalmology and Ocular Oncology, Jagiellonian University Medical College, 31-066 Krakow, Poland; izabasta@gmail.com

**Keywords:** cataract surgery, phacoemulsification, retinal thickness, retinal volume, vascular perfusion, vascular density

## Abstract

*Background*: Cataract phacoemulsification surgery provides excellent refractive results; however, it also elicits changes in the posterior segment of the eye. This study aimed to determine changes in retinal parameters measured by spectral-domain optical coherence tomography (SD-OCT) and OCT angiography (OCTA) after an uncomplicated cataract surgery, including the impact of effective phacoemulsification time (EPT). *Methods*: The study included 44 patients without retinal abnormalities, followed up after unilateral uncomplicated cataract phacoemulsification in a single ophthalmological unit. Patients were evaluated for the following parameters at baseline and at 2 weeks, 3 months, and 12 months after the surgery: best corrected visual acuity, central retinal thickness (CRT), average central retinal thickness (CRTA), central retinal volume (cube volume (CV)), vessel density central (VDC), vessel density full (VDF), vessel perfusion central (VPC), and vessel perfusion full (VPF). The EPT recorded at each procedure was used as a covariant for the evaluation of changes in retinal parameters after the surgery. Analysis included 44 eyes for SD-OCT and 17 for OCTA evaluation, according to adopted scan quality thresholds. *Results*: A significant increase in CRT, CRTA, and CV was noted at each follow-up point compared with baseline. The rising tendency was observed in the first 3 months after the surgery, with a decline over the subsequent months. The VPF parameter showed a stable improvement after the surgery. The analysis of covariance did not confirm any significant effect of the EPT on variations in CRT, CV, CRTA, VDC, and VPF and there was a weak effect on the VDF parameter. *Conclusions*: Uncomplicated cataract surgery results in an increase in retinal thickness and volume in the first few months after the surgery, followed by a spontaneous decline in these parameters in the subsequent months. A long-standing improvement is noted in the VPF parameter.

## 1. Introduction

Cataract surgery is a common routine procedure in ophthalmology. Its effects are usually evaluated in the context of postoperative refraction and visual improvement. However, even uncomplicated cataract extraction may alter the normal retina by inducing an inflammatory process, functional hyperemia, and changes in ocular perfusion pressure [1,2,3,4,5,6]. Information on the intensity and persistence of these alterations could be valid in the context of possible functional improvements but also potential risks (such as the development of retinal disorders).

Variations in retinal thickness after an uncomplicated cataract surgery have been already assessed in the ophthalmological literature [7,8,9,10,11,12,13,14,15,16,17,18,19]. Most studies used optical coherence tomography (OCT) to detect alterations in retinal thickness and the incidence of cystoid macular edema. The advent of OCT angiography (OCTA) has made it possible to additionally assess the effect of cataract surgery on retinal vasculature and perfusion. However, current knowledge on this topic is rather limited, as only a few studies have been completed. Nonetheless, changes in vessel density and retinal perfusion after cataract operation are consistently reported [20,21,22,23,24,25].

The aim of our study was to determine alterations in retinal parameters measured by spectral-domain OCT (SD-OCT) and OCTA after uncomplicated cataract surgery, including the impact of effective phacoemulsification time (EPT).

## 2. Materials and Methods

This retrospective study was conducted in the Department of Ophthalmology of the Specialist Hospital in Chojnice, Poland, between July 2020 and November 2020. We enrolled consecutive patients who had undergone an uncomplicated unilateral cataract surgery and provided written informed consent to participate in the study. Of the 80 included patients, 26 did not complete all the scheduled follow-up visits and 10 cases were excluded due to poor quality of SD-OCT scans.

The inclusion criteria were as follows: consent to participate in the study, a history of uncomplicated cataract surgery, absence of any eye diseases that could affect the retinal architecture (e.g., diabetic retinopathy, macular edema, vitreoretinal interface disorders), the quality of scans of at least 6/10 for SD-OCT and 9/10 for OCTA on the device scale for every examination, and participation in at least three follow-up visits at 2 weeks, 3 months, and 1 year. Additionally, patients with corneal edema and/or posterior capsule opacification were excluded from the study to eliminate bias of poor transparency of the optical media on the measurements. High myopia cases with refractive error greater than or equal to -6D or axial length greater than or equal to 26 mm were also excluded [26,27]. Originally, the study design included also a follow-up visit at 6 months, but this could not be scheduled in all cases due to the COVID-19 lockdown.

The final sample included 44 patients (20 men and 24 women) for the SD-OCT analysis and a subgroup of 17 patients (10 men and 7 women) for the OCTA assessment. The age of patients ranged from 45 to 83 years (mean age, 68.6 ± 12.57 years). Mean spherical equivalent of preoperative refraction was 0.1 ± 2.5D and mean axial length was 23.50 ± 1.08 mm. All patients underwent an uncomplicated cataract surgery with the use of Stellaris (Bausch & Lomb, Bridgewater, NJ, USA) in a micro-coaxial mode and 2.2-mm incision with the implantation of the same type of monofocal intraocular lens (Idea by Croma-Pharma). After each operation, the EPT was recorded for further analysis. The EPT in the Stellaris machine reflects the amount of effective ultrasound power used to treat a single cataract. It is calculated as the multiplication of the arithmetic mean of the power used in the surgery by the total time of phacoemulsification.

After the surgery, each patient received the same regimen of topical treatment: diclofenac sodium (1 mg/mL 3 times daily for 4 weeks; Dicloabak, Laboratoires Thea, Clermont-Ferrand, France), levofloxacin hemihydrate (5 mg/1 mL 4 times daily for 2 weeks; Oftaquix, Santen UK Limited, Tampere, Finland), and dexamethasone phosphate (1 mg/1 mL 4 times daily for 2 weeks; Dexafree, Laboratoires Thea).

**Figure 1:**
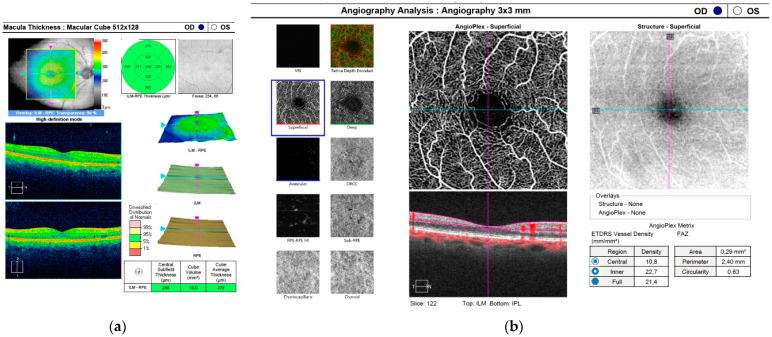
(**a**) Printout of the protocol used for central retinal thickness (CRT), central retinal volume (CV), and average central retinal thickness (CRTA) measurements. (**b**) Printout of the protocol for vessel density measurements.

The SD-OCT and OCTA measurements were performed at all follow-up time points and included the following parameters: central retinal thickness (CRT), average central retinal thickness (CRTA), central retinal volume (cube volume (CV)), vessel density central (VDC), vessel density full (VDF), vessel perfusion central (VPC), and vessel perfusion full (VPF). Examples of printouts of protocols used for SD-OCT and OCTA measurements are presented in Figure 1a,b.

BCVA was tested at each visit on the Snellen chart, and the results were subsequently converted to logMAR units. The applied correction was based on the automated refraction measured with the automated refractor (Huvitz HRK-1, Dongan-gu, Korea, 2018).

SD-OCT examination was performed using the Zeiss AngioPlex SD-OCT (Carl Zeiss Meditec AG, Jena, Germany), which enables the OCT angiography measurements of vessel density and perfusion. In this particular device, the Early Treatment of Diabetic Retinopathy grid is used to determine the measured areas of the central retina. CRT refers to the mean retinal thickness within the central part of the macula of 1 mm in diameter. CRTA corresponds to the mean retinal thickness of the central retinal area of 6 mm in diameter. CV reflects the retinal volume within the central retinal circle of 6 mm in diameter, which approximately refers to the macular area. Vessel density and perfusion density are parameters introduced in the AngioPlex device for the vasculature of the retinal superficial capillary plexus. The scans are obtained from the central retinal square area of 3 mm × 3 mm. Vessel density is defined as the total length of perfused vasculature per unit area and expressed as mm/m^2^. On the other hand, perfusion density measures the percent total area of perfused vasculature in a given region of measurement. In addition to the length, it also involves the caliber of the vessels. Perfusion density is a relation of the number of pixels with perfused vasculature to the total number of pixels in the region; thus, it is expressed in %. Central vessel and perfusion densities (VDC and VPC, respectively) represent the parameters in the central circle of 1 mm in diameter. On the other hand, full vessel and perfusion densities (VDF and VPF, respectively) refer to the central area of 3 mm in diameter.

The study was approved by a local bioethics committee (Komisja Bioetyczna at OIL at OIL in Gdańsk; approval no.: KB-18/20 dated 7 July 2020).

### Statistical Analysis

Statistical analysis was performed using the IBM SPSS Statistics 27 software. (IBM, Armonk, NY, USA) The effects of cataract surgery on retinal parameters were assessed by the repeated measures analysis of variance (ANOVA) for the four time points with the inclusion of the EPT covariant (ANCOVA). The analysis included the following parameters: BCVA, CRT, CRTA, and CV. Each of these parameters was assessed with the same ANCOVA model, and an additional ANOVA Friedman rank test was performed to confirm the differences in cases with high measurement errors. Multipair comparisons were performed with the Bonferroni correction. Statistical significance was reported for a *p* value of 0.05 or lower. OCTA measurements VDC, VDF, VPC, and VPF were evaluated with linear analysis of variance (linear ANCOVA) due to the relatively small sample.

## 3. Results

The descriptive statistics of the study group with the Shapiro–Wilk test results for the normality of distribution (*p* values) are presented in Table 1.

### 3.1. Best Corrected Visual Acuity

There was a significant improvement in BCVA after cataract surgery. The improvement was noted at each follow-up visit (*p* < 0.001). An increasing tendency was maintained at 2 weeks and 3 months, and then the BCVA values remained stable at 1 year after the surgery. There was no difference in BCVA at 3 months and 1 year (*p* = 1.000). The EPT did not significantly affect changes in BCVA during follow-up (ANCOVA F(1.42) = 0.21; *p* = 0.649; η^2^ = 0.01).

### 3.2. Central Retinal Thickness

**Figure 2:**
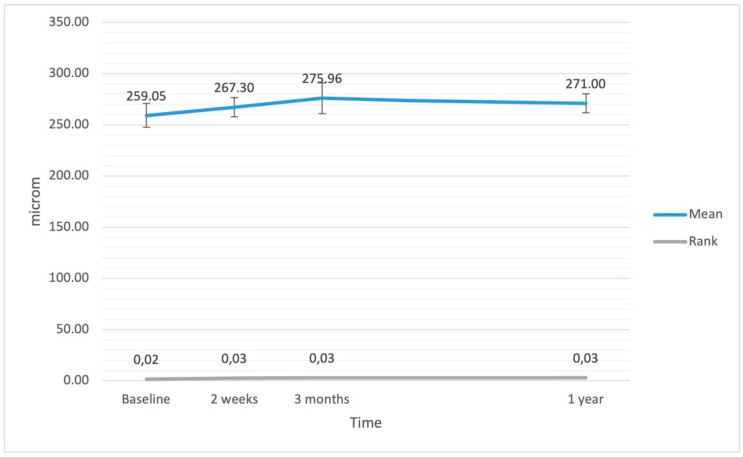
Central retinal thickness (CRT) at baseline and after the surgery. The error bars represent 95% CI of the obtained results.

The ANCOVA assessing changes in CRT after the surgery revealed a result of borderline significance (F(3.126) = 2.55; *p* = 0.086; η^2^ = 0.06). However, the additional ANOVA Friedmann rank test confirmed significant changes (F = 24.49; df = 3; *p* < 0.001). The analysis of simple effects with the Bonferroni correction showed that baseline CRT was significantly lower compared with values at 2 weeks (*p* = 0.028), 3 months (*p* < 0.001), and 1 year (*p* < 0.001). There were no significant differences between CRT values at different follow-up time points after the surgery. Variations in CRT after the surgery are presented in Figure 2.

The ANCOVA did not confirm a relationship between EPT and variations in CRT (F(1.42) = 0.35; *p* = 0.557; η^2^ = 0.01).

### 3.3. Macular Volume

**Figure 3:**
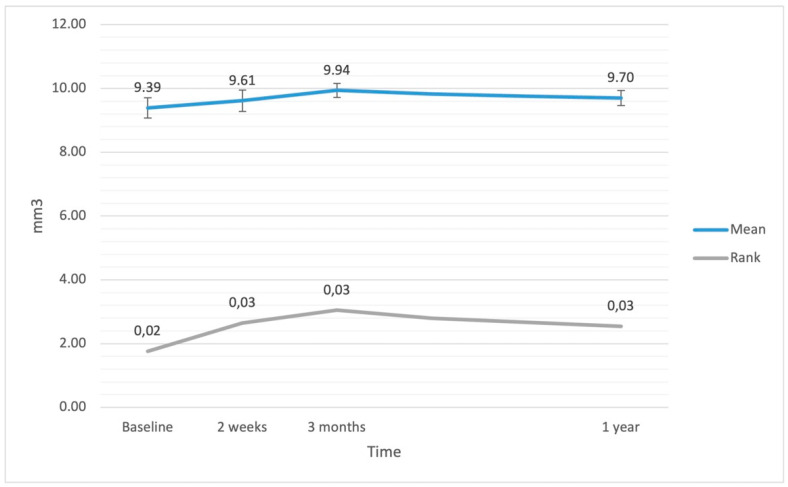
Cube volume (CV; retinal volume within central 6 mm circle) at baseline and after the surgery. The error bars represent 95% CI of the obtained results.

The ANCOVA showed a significant increase in CV only at 3 months after the surgery (*p* = 0.003). However, the additional Friedmann rank test revealed significant changes in CV values at each time point (F = 24.68; df = 3; *p* < 0.001), with a *p* value of 0.008 at 2 weeks, <0.001 at 3 months, and 0.028 at 1 year. The ANCOVA did not show a relationship between EPT and variations in CV (F(1.42) = 0.03; *p* = 0.863; η^2^ < 0.01). Variations in CV after the surgery are presented in Figure 3.

### 3.4. Average Central Retinal Thickness

**Figure 4:**
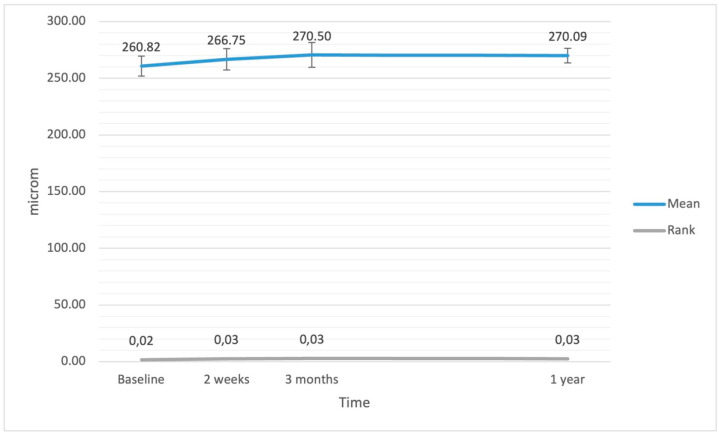
Average central retinal thickness (CRTA) at baseline and after the surgery. The error bars represent 95% CI of the obtained results.

The ANCOVA did not reveal significant variations in CRTA after the surgery, but significance was revealed by the Friedmann rank test (F = 20.48; df = 3; *p* < 0.001). CRTA increased at 2 weeks (*p* = 0.012), 3 months (*p* < 0.001), and 1 year (*p* = 0.020) after the surgery, although the effect was weak. The EPT had no impact on variations in CRTA (F(1.42) < 0.01; *p* = 0.999; η^2^ < 0.01). Variations in CRTA after the surgery are presented in Figure 4.

### 3.5. Vessel Density Central

**Figure 5:**
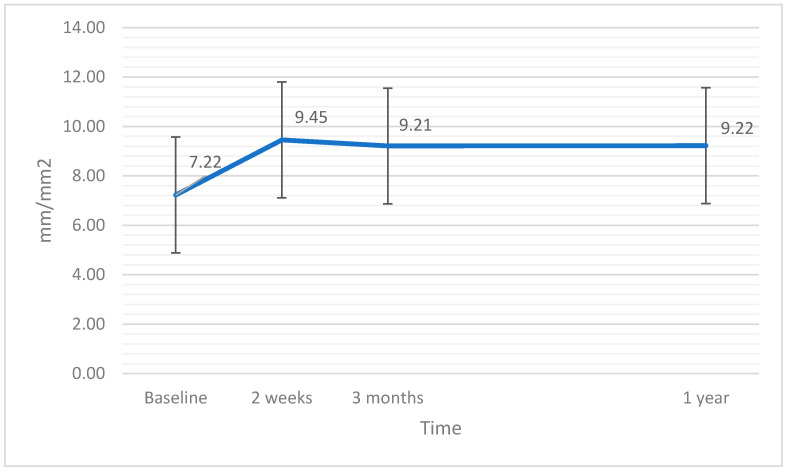
Vessel density in the central part of the retina (VDC) at baseline and after the surgery. The error bars represent 95% CI of the obtained results.

The linear ANCOVA test did not reveal significance of variations in VDC after the surgery (F(3,60) = 0.26; *p* = 0.857; η^2^ = 0.04), nor was the significant effect of the covariant of EPT time proven (*p* = 0.243). Variations in VDC after the surgery are presented in Figure 5.

### 3.6. Vessel Density Full

**Figure 6:**
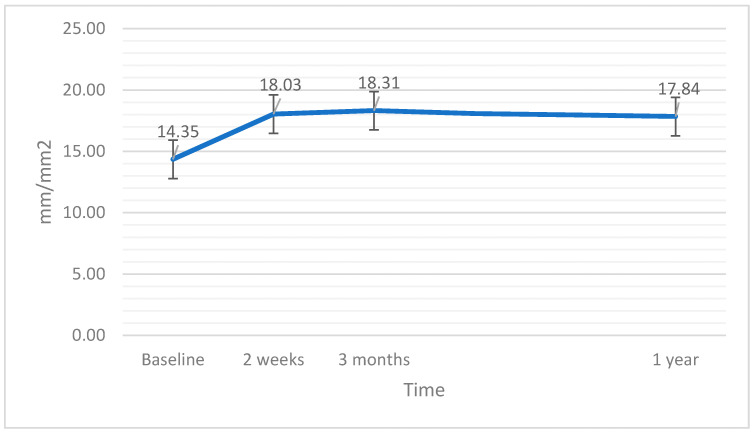
Vessel density in the larger central part of the retina (VDF) at baseline and after the surgery. The error bars represent 95% CI of the obtained results.

Analysis of variations in the VDF parameter after the surgery with the covariant of EPT did not show statistical significance (F(3,60) = 2,21; *p* = 0.096; η^2^ = 0.20). However there was a weak relationship between higher EPT values and an increase in VDF (F(3,60) = 4.20; *p* = 0.045; η^2^ = 0.36; r = 0.22). Variations in VDF after the surgery are presented in Figure 6.

### 3.7. Vessel Perfusion Central

**Figure 7:**
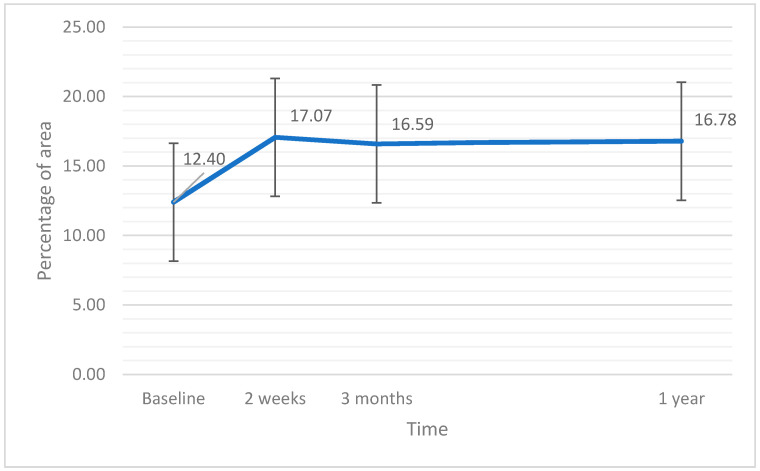
Perfusion at the central part of the retina (PVC) at baseline and after the surgery. The error bars represent 95% CI of the obtained results.

The linear ANCOVA analysis did not show significant variations in VPC after the operation (F(3,60) = 0.42; *p* = 0.741; η^2^ = 0.05), nor was the effect of the covariant of EPT time revealed (*p* = 0.277). Variations in VPC after the surgery are presented in Figure 7.

### 3.8. Vessel Perfusion Full

**Figure 8:**
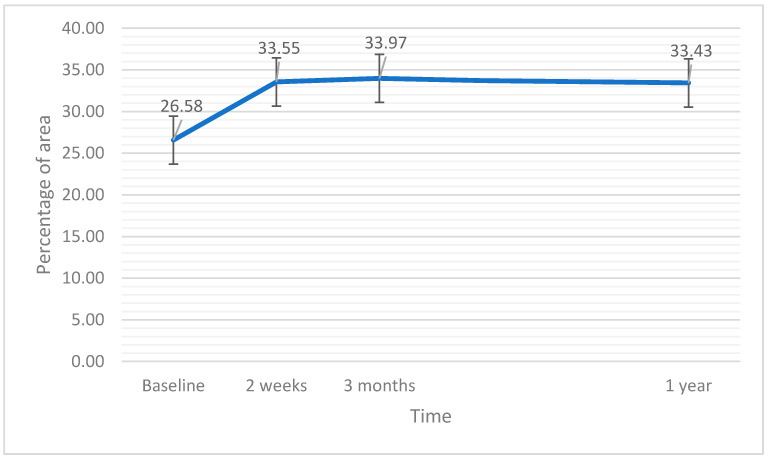
Vessel perfusion full (VPF) at baseline and after the surgery. The error bars represent 95% CI of the obtained results.

The linear ANCOVA test revealed significant variations in VPF after the surgery (F(3,60) = 2.98; *p* = 0.038; η^2^ = 0.21). Nevertheless, there was no covariant effect of EPT (*p* = 0.106). The analysis of simple effects with Bonferroni correction showed a significant increase in VPF at each follow-up time point (*p* < 0.05). No significant differences among post-surgical VPF measurements were noted (*p* = 1.0). Variations in VPF after the surgery are presented in Figure 8.

## 4. Discussion

### 4.1. Summary of the Results

In our study group, BCVA improved significantly after the surgery. BCVA achieved normal values in almost all patients (mean 0.02 ± 0.06 logMAR) at 3 months after the surgery, and the effect was maintained at 12 months (mean 0.01 ± 0.03 logMAR).

The statistical analysis confirmed significant postoperative changes in retinal thickness. CRT was increased at each follow-up time point compared with baseline. CV showed a significant increase at 3 months in the ANOVA and an increase at all three follow-up time points in the Friedmann rank test. CRTA also increased significantly at all three time points after the surgery, although the effect had moderate power. The increase in retinal thickness and volume had a tendency to rise in the first few months after the surgery and then to drop in the subsequent months. Nevertheless, the values at 12 months were still higher compared with baseline.

There were no significant changes in variations in VDC, VDF, and VDC after the surgery. On the other hand, vascular perfusion in the full retinal area (3 mm^2^) improved after treatment. The increase was noted already at 2 weeks after the surgery and was maintained without fluctuations at subsequent time points. The question of why the improvement in vascular perfusion after the surgery was noted at the larger central retinal area but not in the smaller central part of 1 mm^2^ has to be addressed. It should be remembered that retinal vascularity in the central 1 mm^2^ naturally presents lower vessel density due to the presence of the foveal avascular zone; thus, changes in perfusion or vascular network density are more difficult detect [28]. This is why a larger sample is probably needed to confirm the perfusion improvement in this area.

### 4.2. SD-OCT and OCTA Measurements

When interpreting SD-OCT and OCTA measurements, the technical limitations of the procedure have to be considered. In our study, the retinal thickness and vascular perfusion parameters at baseline were lower than those after the surgery. Obviously, the presence of cataract affects the quality of the scans. Therefore, it is necessary to address the possible bias resulting from the poorer quality of presurgical scans. The ANOVA showed variations in scan quality, but the effect had moderate statistical power.

According to the manufacturer’s recommendations, the threshold for signal strength accepted in our study (6/10) is also acceptable for the analysis of SD-OCT macular scans [29,30]. Previous studies showed that poorer quality of OCT scans may affect the outcome of the measurements, even if the signal strength or quality index stays above the threshold indicated by the device provider. However, these reports usually refer to optic nerve evaluation [31,32]. There is evidence showing that, in the case of macular cube scans, the lower quality affects the measurements to a lower degree [33]. Therefore, considering these reports, as well as the high mean and median values of signal strength in our study, we believe that the bias of low-quality scans for SD-OCT measurements can be treated as insignificant.

The issue of image quality is especially important for OCTA evaluation, as OCTA is burdened with the problem of artifacts. Most reports confirmed the impact of poorer-quality OCTA scans on the measurement of vascular parameters, especially for repeated measurements [34,35,36]. Some authors directly recommend OCTA scan quality as high as 9/10 for comparative analysis [37]. In order to avoid bias of poor scan quality, we adopted the same high threshold for OCTA analysis.

### 4.3. EPT Time and Results after the Surgery

The ANCOVA revealed the significant effect of the EPT only on variations in VDF and not on any other retinal parameter. Additionally, this positive EPT effect was weak in power, so, taking into account the small sample of patients in this analysis, it needs to be confirmed in a larger study. In our study, cataract was not graded according to its density before treatment. Therefore, considering the acceptance of only high-quality OCT scans for the study, it can be assumed that hard and dense cataracts were excluded.

### 4.4. Other Studies

Our results should be placed within the context of other studies. Notably, our study has the strength of a long follow-up, which was 12 months. Studies with a follow-up of 6 months and longer reported similar results. A transient increase in CRT was typically observed after cataract surgery, with a peak value at around 2–3 months, followed by a drop to lower or baseline values [13,15,16,17,18,20]. In our study, CRT at the end of follow-up was still higher than before cataract phacoemulsification.

There are scarce data on changes in OCTA parameters after cataract extraction (vascular density and vascular perfusion), with only a few studies available [20,21,22,23,24,25]. In a large study of 55 eyes, Krizanovic et al. [20] reported a stable improvement in vessel length and area, which was most prominent in the superficial vascular complex, noted from week 1 through month 3 after cataract extraction. Similarly, Zhao et al. [22] showed a significant increase in vascular density at 3 months after treatment in a study of 32 eyes. Zhou et al. [25] revealed a significant improvement in macular and peripapillary vascular parameters in a 1-month study of 51 eyes. The increase was noted after 1 week from surgery. Feng et al. [24] described an increase in superficial capillary plexus parameters in diabetic patients (32 eyes) 3 months after the surgery, but not in patients without diabetes. On the other hand, a small study of nine eyes by Pilotto et al. [21] showed only a transient increase in OCT and OCTA parameters, with a return to baseline at day 90 after the surgery. Interestingly, Li et al. [23] reported an improvement in vascular parameters 3 months after the surgery in patients with low myopia (31 eyes) and a worsening in patients with high myopia (24 eyes).

The impact of phaco energy during cataract surgery on retinal thickness parameters has not been widely investigated. Kurt et al. [12] reported a positive correlation between EPT and an increase in ganglion cell layer (GCL) and inner nuclear layer (INL) thickness. Similarly, Gołębiowska et al. [16] described higher values of retinal thickness and volume with higher phaco power used during the surgery, but not with longer phaco time. Other studies did not confirm the correlation between intraoperative factors (phaco energy, phaco duration) and retinal thickening after the surgery [8,17].

Thus far, the relationship between changes in vascular retinal parameters and the amount of phaco energy used during the procedure has been assessed only in the short-term study by Zhou et al. [25]. The authors reported an increase in vessel density and perfusion density with larger values of cumulative dissipated phaco energy used during the procedure. However, they did not provide an explanation of the cause of this phenomenon. In our study, we found only a weak correlation between a higher EPT and VDF, and not with any other vascular retinal parameters. As our sample for the OCTA analysis was relatively small, this finding has to be interpreted with caution and confirmed in a larger trial.

An additional issue that should be taken into consideration when analyzing the impact of cataract surgery on postoperative retinal parameters is the type of pump implemented in the phaco machine. To our knowledge, this issue has not been addressed in the medical literature so far; therefore, only theoretical considerations can be presented. The two main types of pumps used in devices for cataract surgery are the Venturi pump and the peristaltic pump. A few available comparative studies showed that the Venturi pump requires smaller energy levels and a shorter time to complete cataract surgery than the peristaltic pump [38,39]. The Venturi type is also associated with a lower incidence of thermal corneal burns related to excessive thermal energy levels during the surgery [39]. The Stellaris phaco machine used in our study utilizes the Venturi pump; thus, we employed the technical option that was less likely to produce excessive energy levels during the surgery. If the higher phaco energy used during cataract surgery were to be related to postoperative retinal vascular changes, then it would be more likely to occur with the use of the peristaltic pump. However, this issue requires further comparative studies.

### 4.5. The Pathophysiological Concepts

The increase in retinal thickness and improvement in vascular parameters after cataract surgery could be attributed to a few processes. First, it could be due to the inflammatory reaction triggered by the surgery itself and hampered routinely by the application of topical anti-inflammatory drugs, as widely described in the literature [40,41]. Typically, inflammation after cataract surgery reaches its peak within a few days postoperatively and then declines over 2–3 weeks [42]. Our study showed that variations in retinal parameters are long-standing and, as such, they are quite unlikely to result from an inflammatory process induced by surgery. Moreover, none of the patients included in the study demonstrated any signs of chronic inflammation during the follow-up, including cystoid macular edema. The second postulated mechanism is associated with a decrease in intraocular pressure noted after cataract surgery, which could improve retinal perfusion and increase OCTA parameters [43,44,45]. While we did not address this issue in our study, other authors who reported postsurgical improvements in retinal vessels did not identify a relationship between a reduction in intraocular pressure and an increase in ocular perfusion pressure [20,25]. The third mechanism underlying the vascular improvement after cataract surgery is functional hyperemia, which is induced by an increase in light exposure leading to enhanced retinal metabolism [5,46]. Increased retinal metabolism involves the consumption of large oxygen and glucose amounts, which triggers the production of vasoactive mediators leading to vasodilation and hyperemia [6]. Logically, this effect should be reflected in an increase in OCTA parameters. According to our results, the improvement in retinal perfusion after cataract surgery is stable and long-standing, which indicates the third mechanism as the most plausible.

Nevertheless, it is important to note that despite an improvement in vascular perfusion, the higher exposure to light can also trigger angiogenesis, especially in susceptible eyes with signs of age-related macular degeneration. Further research on specific subgroups of patients is needed to establish whether the benefits of enhanced light exposure after cataract extraction outweigh the potential risks.

### 4.6. Study Limitations

The study included a relatively small number of cases, especially in the OCTA analysis, and its results have to be confirmed by large clinical trials. However, most current OCTA-based studies on this topic include a relatively small number of subjects. A dditionally, as dense cataract significantly reduces the quality of OCTA scans, the issue of applying the results to these cases remains to be clarified. Finally, the decrease in intraocular pressure after the surgery was not assessed as a potential factor influencing the improvement in vascular retinal parameters.

## 5. Conclusions

Uncomplicated cataract surgery in patients without retinal diseases results in an increase in retinal thickness and volume, with a peak value at around 3 months after the surgery and a subsequent decline. Vascular perfusion in the central 3 mm^2^ of the macular area assessed by OCTA shows a stable improvement after the surgery. The EPT has no apparent impact on the variations in most of the retinal parameters after cataract surgery.

## Figures and Tables

**Table 1:** Results for visual acuity, effective phacoemulsification time, and SD-OCT and OCTA measurements before and after the surgery.

Parameter	Mean	Median	SD	*p*
Phacoemulsification time				
effective (EPT)	2.95	2.35	3.44	<0.001
At baseline				
best corrected visual acuity (logMAR)	0.57	0.50	0.34	0.001
central retinal thickness (μm)	259.05	262.00	38.94	<0.001
central volume (mm^3^)	9.39	9.65	1.07	<0.001
average retinal thickness (μm)	260.82	267.50	29.73	<0.001
vascular density central (mm/mm^2^)	7.22	5.1	5.29	0.071
vascular density full (mm/mm^2^)	14.35	13.90	4.05	0.969
vascular perfusion central (%)	12.40	9.30	9.34	0.038
vascular perfusion full (%)	26.58	25.80	8.16	0.556
2 weeks after the surgery				
best corrected visual acuity (logMAR)	0.10	0.00	0.13	<0.001
central retinal thickness (μm)	267.30	259.00	31.80	0.010
central volume (mm^3^)	9.61	9.90	1.12	<0.001
average retinal thickness (μm)	266.75	275.50	31.28	<0.001
vascular density central (mm/mm^2^)	9.45	8.80	4.77	<0.001
vascular density full (mm/mm^2^)	18.03	18.70	2.66	<0.001
vascular perfusion central (%)	17.06	15.60	8.83	0.003
vascular perfusion full (%)	33.55	33.70	4.69	<0.001
3 months after the surgery				
best corrected visual acuity (logMAR)	0.02	0.00	0.06	<0.001
central retinal thickness (μm)	275.95	271.00	50.97	<0.001
central volume (mm^3^)	9.94	10.00	0.72	0.753
average retinal thickness (μm)	270.50	273.00	36.32	<0.001
vascular density central (mm/mm^2^)	9.21	8.30	4.61	0.218
vascular density full (mm/mm^2^)	18.31	19.40	3.38	0.007
vascular perfusion central (%)	16.59	14.60	8.33	0.107
vascular perfusion full (%)	33.97	35.50	5.63	0.025
1 year after the surgery				
best corrected visual acuity (logMAR)	0.01	0.00	0.03	<0.001
central retinal thickness (μm)	271.00	272.00	31.26	0.198
central volume (mm^3^)	9.70	9.80	0.79	0.011
average retinal thickness (μm)	270.09	273.00	21.79	0.017
vascular density central (mm/mm^2^)	8.40	8.40	4.68	0.418
vascular density full (mm/mm^2^)	17.84	18.40	3.25	0.209
vascular perfusion central (%)	16.78	14.70	8.53	0.240
vascular perfusion full (%)	33.43	35.30	5.37	0.142

## Data Availability

Data are available from the corresponding author upon request.
